# Draft Genome Sequence of *trh*^+^ *Vibrio parahaemolyticus* VP-49, Isolated from Seafood Harvested along the Mangalore Coast, India

**DOI:** 10.1128/genomeA.00607-14

**Published:** 2014-06-26

**Authors:** Ballamoole Krishna Kumar, Vijaya Kumar Deekshit, Praveen Rai, Volker Gurtler, Iddya Karunasagar, Indrani Karunasagar

**Affiliations:** a UNESCO-MIRCEN for Marine Biotechnology, Department of Fisheries Microbiology, Karnataka Veterinary, Animal and Fisheries Sciences, University, College of Fisheries, Mangalore, India; b School of Applied Sciences, Bundoora Campus RMIT University, Bundoora, Australia; c FAO Consultant, Subba-Meena, Jayanagar, Mangalore, India

## Abstract

*Vibrio parahaemolyticus* is a seafood-borne pathogen autochthonous to the marine and estuarine ecosystem, which is responsible for gastroenteritis due to the consumption of contaminated raw seafood. Here, we report the draft genome sequence of *V. parahaemolyticus* VP-49, isolated from seafood, to identify the different virulence attributes and to study the mechanisms that enhance its environmental fitness.

## GENOME ANNOUNCEMENT

*Vibrio parahaemolyticus* is a Gram-negative, halophilic bacterium autochthonous to the marine environments and is responsible for causing seafood-borne gastroenteritis in humans (1–4). Recently, we have identified and analyzed the distribution of the T3SS2 genes in *trh*^+^ *V. parahaemolyticus* isolated from seafood harvested along the Mangalore coast, India (5). Here, we report the draft genome sequence of one such isolate of *V. parahaemolyticus*, named VP-49, with the aim of understanding the distinction between clinical and environmental isolates of this versatile pathogen.

Genomic DNA was extracted from *V. parahaemolyticus* VP-49 using a QIAamp DNA minikit (Qiagen, Germany). A concentration of 50 ng/µl was used for the genome sequencing. The raw sequence data were generated after library preparation on the Ion Torrent PGM platform. The sequence data were assembled using CLC Genomics Workbench version 6. Structural gene prediction and functional annotation were performed using the Rapid Annotations using Subsystems Technology (RAST) server (6).

A total of 1,017,077 reads with a mean read length of 160 bp for 200-bp fragmentation chemistry obtained from the Ion PGM were assembled into 137 contigs. The draft genome had a length of 5,047,822 bp, as expected for *V. parahaemolyticus* (7), with a total of 4,643 genes. The 137 contigs from VP-49 were also assembled to UCM-V493 (an environmental isolate negative for *tdh* and *trh* that lacks all 7 pathogenicity islands [8]) using Geneious 6.1.6. The ribosomal RNA operon (*rrn*) genomic organization for isolate VP-49 was identical to that of isolate UCM-V493, lending further support to the theory that they are closely related as environmental isolates even though they have different *tdh* and *trh* genotypes. This was shown when the contigs assembled to all parts of the UCM-V493 genome except to the repetitive *rrn* sequences. These gaps were not only confirmed to contain *rrn* operon sequences (9, 10), but by using DNA sequences flanking the 3′ region of the 16S rRNA gene and the 5′ region of the 5S rRNA gene, allele-specific DNA sequences were identified for each operon and the chromosomal location of each operon was confirmed to be identical between strains. Furthermore, each strain had 9 *rrn* operons, 8 on chromosome I and 1 on chromosome II.

The analysis obtained from the RAST server revealed 533 subsystems. The annotated genome has 74 genes responsible for resistance to antibiotic and toxic compounds, including 26 genes for multidrug resistance efflux pumps. A total of 238 genes code for membrane transport proteins. A brief analysis of the *V. parahaemolyticus* VP-49 draft genome also revealed the presence of genes coding for thermostable direct hemolysin (TDH)-related hemolysins (T3SS1 and T3SS2) and showed that this strain lacks unique sequences found in the acute hepatopancreatic necrosis disease (AHPND)-causing *V. parahaemolyticus*. The T3SS2 of VP-49 had a G+C content of 38.2%, which is considerably lower than the rest of the genome, which is characteristic of the pathogenicity island acquired through horizontal gene transfer. The draft genome sequence of *V. parahaemolyticus* VP-49 will be useful in future studies to determine the different virulence attributes as well as mechanisms that enhance its environmental or host fitness.

### Nucleotide sequence accession number.

This whole-genome shotgun project has been deposited at DDBJ/EMBL/GenBank under the accession number JEMS00000000. The version described in this paper is the first version.
